# Wedelolactone Acts as Proteasome Inhibitor in Breast Cancer Cells

**DOI:** 10.3390/ijms18040729

**Published:** 2017-03-29

**Authors:** Tereza Nehybová, Jan Šmarda, Lukáš Daniel, Marek Stiborek, Viktor Kanický, Ivan Spasojevič, Jan Preisler, Jiří Damborský, Petr Beneš

**Affiliations:** 1 Laboratory of Cell Differentiation, Department of Experimental Biology, Faculty of Science, Masaryk University, Kamenice 5, 625 00 Brno, Czech Republic; 322903@mail.muni.cz (T.N.); smarda@sci.muni.cz (J.Š.); 2 International Clinical Research Center, Center for Biological and Cellular Engineering, St. Anne’s University Hospital, Pekarska 53, 656 91 Brno, Czech Republic; 211165@mail.muni.cz (L.D.); jiridamborsky0@gmail.com (J.D.); 3 Loschmidt Laboratories, Department of Experimental Biology and Research Centre for Toxic Compounds in the Environment RECETOX, Faculty of Science, Masaryk University, Kamenice 5, 625 00 Brno, Czech Republic; 4 Department of Chemistry, Masaryk University, Kamenice 5, 625 00 Brno, Czech Republic; 408516@mail.muni.cz (M.S.); viktor.kanicky@ceitec.muni.cz (V.K.); preisler@chemi.muni.cz (J.P.); 5 CEITEC-Central European Institute of Technology, Masaryk University, Kamenice 5, 625 00 Brno, Czech Republic; 6 Department of Life Sciences, Institute for Multidisciplinary Research, University of Belgrade, 11030 Belgrade, Serbia; redoxsci@gmail.com

**Keywords:** breast cancer, copper, proteasome, reactive oxygen species, wedelolactone

## Abstract

Wedelolactone is a multi-target natural plant coumestan exhibiting cytotoxicity towards cancer cells. Although several molecular targets of wedelolactone have been recognized, the molecular mechanism of its cytotoxicity has not yet been elucidated. In this study, we show that wedelolactone acts as an inhibitor of chymotrypsin-like, trypsin-like, and caspase-like activities of proteasome in breast cancer cells. The proteasome inhibitory effect of wedelolactone was documented by (i) reduced cleavage of fluorogenic proteasome substrates; (ii) accumulation of polyubiquitinated proteins and proteins with rapid turnover in tumor cells; and (iii) molecular docking of wedelolactone into the active sites of proteasome catalytic subunits. Inhibition of proteasome by wedelolactone was independent on its ability to induce reactive oxygen species production by redox cycling with copper ions, suggesting that wedelolactone acts as copper-independent proteasome inhibitor. We conclude that the cytotoxicity of wedelolactone to breast cancer cells is partially mediated by targeting proteasomal protein degradation pathway. Understanding the structural basis for inhibitory mode of wedelolactone might help to open up new avenues for design of novel compounds efficiently inhibiting cancer cells.

## 1. Introduction

The ubiquitin-proteasome system (UPS) controls a highly complex and tightly regulated process of cellular protein degradation. In contrast to rather non-specific extracellular/membrane protein degradation by lysosomes, the proteasomes destroy proteins labelled with polyubiquitin chains. UPS consists of numerous protein components (E1, E2, E3 enzymes, proteasome, deubiquitinases). The mammalian cytosolic 26S proteasome complex contains the core 20S proteasome capped with one or two 19S regulatory subunit(s). The proteolytic activities are located in β1, β2, and β5 subunits of the core 20S proteasome complex [1,2,3]. The eukaryotic proteasome possesses at least three distinct protease activities: chymotrypsin-like (cleavage after hydrophobic residues, located in β5 subunit), trypsin-like (cleavage after basic residues, located in β2 subunit), and caspase-like (cleavage after acidic residues, located in β1 subunit) [4]. The chymotrypsin-like activity is usually the strongest one [5].

Deregulation of UPS has severe effect on cellular function and homeostasis. Over 80% of cellular proteins are degraded via UPS including those involved in regulation of cell proliferation, differentiation, immune signaling, and cell response to stress [3,6]. Emerging evidence show that the targeting of UPS degradation pathway might be a viable anticancer strategy. Due to increased rate of genomic mutations, transformed cells accumulate large quantities of misfolded or overexpressed proteins. In response to such accumulation, malignant cells enhance the expression and activity of UPS [5,7]. Preclinical studies have confirmed a higher susceptibility of malignant cells to cytotoxic effects of UPS inhibitors when compared to normal cells [6,8]. Pertinent to this, several proteasome inhibitors have entered clinical trials, and some have already been approved for the treatment of aggressive hematopoietic tumors [9,10]. However, tumor cell resistance and high toxicity remain an issue in solid tumors leading to search for new UPS inhibitors [5].

Wedelolactone, a natural coumestan, is one of the bioactive compounds found in extracts of *Eclipta alba* and *Wedelia calendulacea* [11]. Recently, in vitro and in vivo anti-cancer properties of wedelolactone in solid tumors including breast, colon, prostate, hepatocellular, pituitary cancers, and neuroblastoma were described in a number of reports [12,13,14,15,16,17,18,19]. Wedelolactone is clearly a multi-target compound and its anti-cancer properties were primarily attributed to the inhibition of multiple kinases, androgen receptor, 5-lipoxygenase, and the c-Myc protein [13,15,17,18,19,20,21]. However, it was found recently that wedelolactone also inhibits topoisomerase IIα activity and blocks DNA synthesis in the breast cancer cells, and that these effects are promoted by copper ions, at least partially via redox interactions [12,22].

This study shows that wedelolactone acts as inhibitor of 20S/26S proteasome chymotrypsin-like and to lesser extent also trypsin-like and caspase-like activities. Treatment of breast cancer cells with wedelolactone resulted in accumulation of ubiquitinated proteins and proteins representing typical proteasomal targets, such as p21, p27, p53, and Bax. Molecular docking revealed a productive binding of wedelolactone to the active sites of β1, β2, and β5 proteasomal subunits with a stronger preference for β5 subunit. The proteasome inhibition by wedelolactone is not dependent on cellular copper level in breast cancer cells. This study concludes that wedelolactone acts as copper-independent inhibitor of proteasome.

## 2. Results

### 2.1. Wedelolactone Inhibits Proteolytic Activities of Proteasome in Breast Cancer Cell Lines

**Figure 1:**
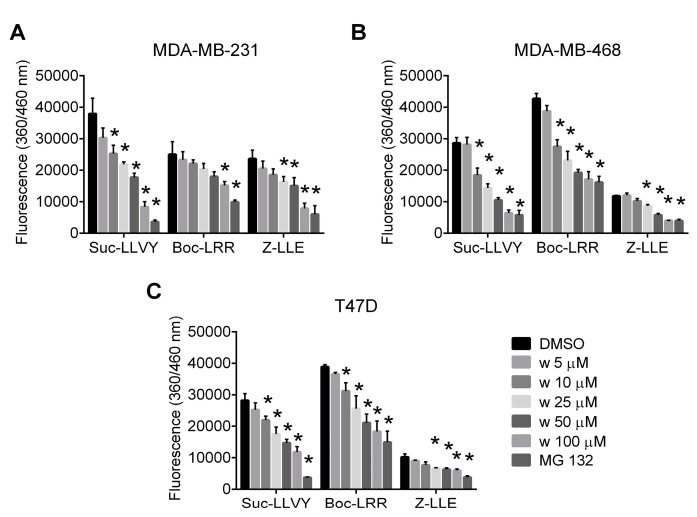
Wedelolactone inhibits chymotrypsin-like, trypsin-like and caspase-like activities in breast cancer cells. MDA-MB-231 (**A**); MDA-MB-468 (**B**); and T47D (**C**) cells were treated with various concentrations of wedelolactone (w) for 10 h. Proteasome activities were evaluated in cell extracts using the activity-specific fluorogenic substrates (Suc-LLVY-AMC for testing chymotrypsin-like, Z-LLE-AMC for caspase-like, and Boc-LRR-AMC for trypsin-like activities). Treatment with MG132 served as a positive control. The data represent the mean values from three independent experiments. Error bars indicate the SD. * indicates a significant (*p* < 0.05) difference between wedelolactone-/MG132- and DMSO-treated cells.

MDA-MB-231, MDA-MB-468, and T47D cells were exposed to increasing concentrations of wedelolactone to study its effect on proteasome in breast cancer cells. Chymotrypsin-like, trypsin-like and caspase-like activities of proteasome were evaluated in cell extracts using the activity-specific fluorogenic substrates. Wedelolactone inhibited all three proteolytic activities of proteasome with the highest potency for the chymotrypsin-like activity (IC_50_ values 27.8 μM for MDA-MB-231, 12.78 μM for MDA-MB-468 and 19.45 μM for T47D) (Figure 1).

### 2.2. Wedelolactone Inhibits Proteolytic Activities of Purified 20S and 26S Proteasome Complexes In Vitro

**Figure 2:**
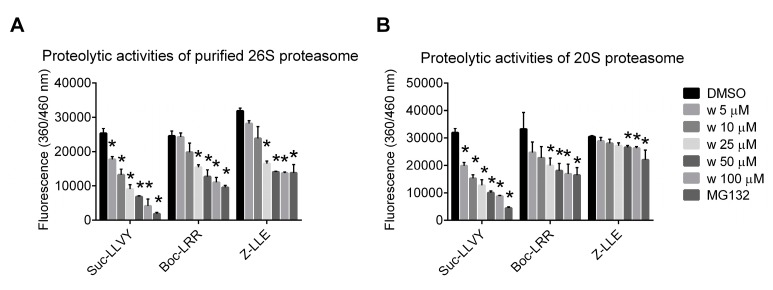
Wedelolactone inhibits chymotrypsin-like, trypsin-like, and caspase-like activities of purified 26S and 20S proteasome complexes in vitro. Wedelolactone (w) was added at various concentrations to reaction mixture containing either (**A**) 26S proteasome purified from MDA-MB-231 cells or (**B**) commercially available 20S proteasome, and fluorogenic substrate (Suc-LLVY-AMC for testing chymotrypsin-like, Z-LLE-AMC for caspase-like, and Boc-LRR-AMC for trypsin-like activities). Fluorescence was measured after 1 h incubation. MG132 was used as a positive control. The data represent the mean values from three independent experiments. Error bars indicate the SD. * indicates a significant (*p* < 0.05) difference in proteolytic activities between reaction mixtures containing wedelolactone/MG132 and DMSO.

The 26S proteasome purified from MDA-MB-231 cells and the commercially available 20S proteasome were incubated separately with the activity-specific fluorogenic substrates and wedelolactone in various concentrations to evaluate the ability of wedelolactone to inhibit their chymotrypsin-like, trypsin-like, and caspase-like activities. Wedelolactone inhibited all three proteasomal activities in vitro in a dose-dependent manner with the highest potency against the chymotrypsin-like activity (IC_50_ values 9.97 μM for 26S and 6.13 μM for 20S proteasome) (Figure 2).

### 2.3. Wedelolactone Causes Accumulation of Polyubiquitinated and Short-Lived Proteins in Breast Cancer Cells

**Figure 3:**
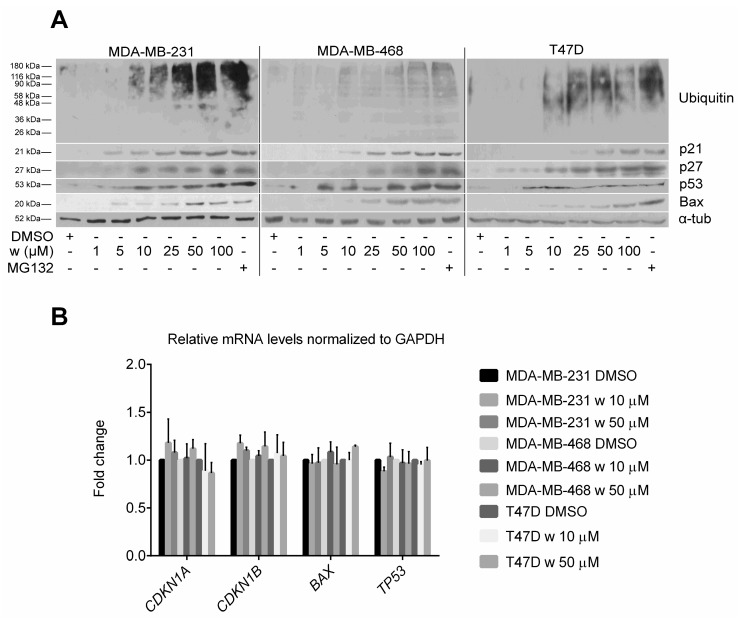
Multiubiquitinated and high turnover proteins accumulate in wedelolactone-treated breast cancer cells. MDA-MB-231, MDA-MB-468, and T47D cells were treated with various concentrations of wedelolactone or solvent for 10 h. (**A**) Protein extracts were subsequently analyzed by SDS-PAGE and immunoblotting using p21-, p27-, p53-, Bax-, and ubiquitin-specific antibodies. Treatment with MG132 served as a positive control; (**B**) Transcripts of *CDKN1A*, *CDKN1B*, *TP53* and *BAX* genes were quantified using qPCR.

The level of polyubiquitinated proteins and p21, p27, p53, and Bax proteins in wedelolactone-treated MDA-MB-231, MDA-MB-468, and T47D breast cancer cells were analyzed by immunoblotting to further confirm the inhibitory effect of wedelolactone on proteasome. We found the dose-dependent accumulation of p21, p27, p53, Bax as well as multiubiquitinated proteins occurring in all three cell lines tested (Figure 3A). To confirm that the wedelolactone-induced accumulation of p21, p27, p53, and Bax proteins is not caused by increased rate of their transcription/expression, the transcripts of corresponding genes were quantified using quantitative polymerase chain reaction (qPCR). We found that wedelolactone did not affect expression of any of these genes in all three breast cancer cell lines (Figure 3B).

### 2.4. Cytotoxicity of Wedelolactone Increases for Cells with High Content of Intracellular Copper

**Figure 4:**
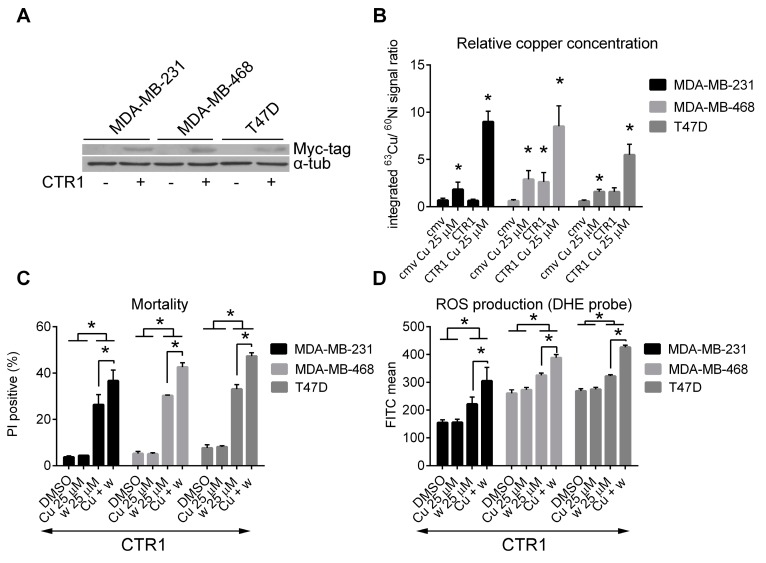
Copper loading enhances cytotoxicity and ROS production in wedelolactone-treated breast cancer cells. MDA-MB-231, MDA-MB-468, and T47D cells were transiently transfected with pCNDA3.1-hCTR1-N-Myc (CTR1) or control pCDNA3.1 plasmids (cmv), pretreated with copper sulfate (Cu) for 24 h and subsequently treated with wedelolactone (w) or solvent (DMSO) in fresh media. (**A**) Cells were harvested and expression of exogenous CTR1 protein was confirmed by SDS-PAGE followed by immunoblotting with the Myc-Tag antibody; (**B**) Relative copper concentration in cell lysates was analyzed by SALD ICP MS. Data for copper are presented as an integrated ^63^Cu/^60^Ni signal ratio. (**C**) Cell mortality and (**D**) ROS production were evaluated after PI/DHE staining using flow-cytometry. The data represent the mean values from three independent experiments. Error bars indicate the SD. * indicates a significant (*p* < 0.05) difference.

The authors of this study suggested previously that cytotoxicity of wedelolactone can be at least partly explained by redox-cycling with copper ions, reactive oxygen species (ROS) generation and promoted oxidative stress [22]. To confirm the role of copper ions in cytotoxicity of wedelolactone, breast cancer cells were transiently transfected with plasmid coding for human copper transporter *CTR1*. The transfection efficiency determined by flow-cytometry was 65.6% ± 3.7%, 43.6% ± 4.2%, and 46.6% ± 3.8% in MDA-MB-231, MDA-MB-468, and T47D cells, respectively (Figure S1). Expression of exogenous CTR1 protein was confirmed by immunoblotting (Figure 4A). Transfected cells were exposed to copper sulfate for 24 h or left untreated. Analysis of relative copper concentrations in cell lysates revealed that only combination of *CTR1* overexpression/copper supplementation efficiently increases copper-loading of all three cell lines (Figure 4B). Therefore, for next set of experiments, copper-loaded cells were prepared by simultaneous *CTR1* overexpression/copper supplementation. Copper-loaded and control cells (both over-expressing exogenous *CTR1*) were exposed to wedelolactone or DMSO for 48 h and their mortality was assessed by PI exclusion assay using flow-cytometry. In agreement with our hypothesis, cytotoxicity induced by wedelolactone was enhanced by copper loading (Figure 4C, Figure S2). Furthermore, to analyze whether copper loading enhances the wedelolactone-induced ROS production, copper-loaded and control cells (both over-expressing exogenous *CTR1*) were treated with wedelolactone or solvent for 10 h and ROS production was analyzed after DHE staining by flow-cytometry. Copper-loaded cells produced more ROS in response to wedelolactone than controls (Figure 4D, Figure S3). No significant differences in cell mortality or ROS production was observed in mock-transfected cells that were either pre-incubated with copper or left untreated and subsequently exposed to wedelolactone (Figure S4). It is hypothesized that intracellular level of copper did not reach the required threshold in this case. These results support previous findings that cytotoxicity of wedelolactone is at least partially mediated via (redox) interactions with copper ions.

### 2.5. Copper Does Not Affect the Inhibition of Proteasome Activity by Wedelolactone in Breast Cancer Cell Lines

**Figure 5:**
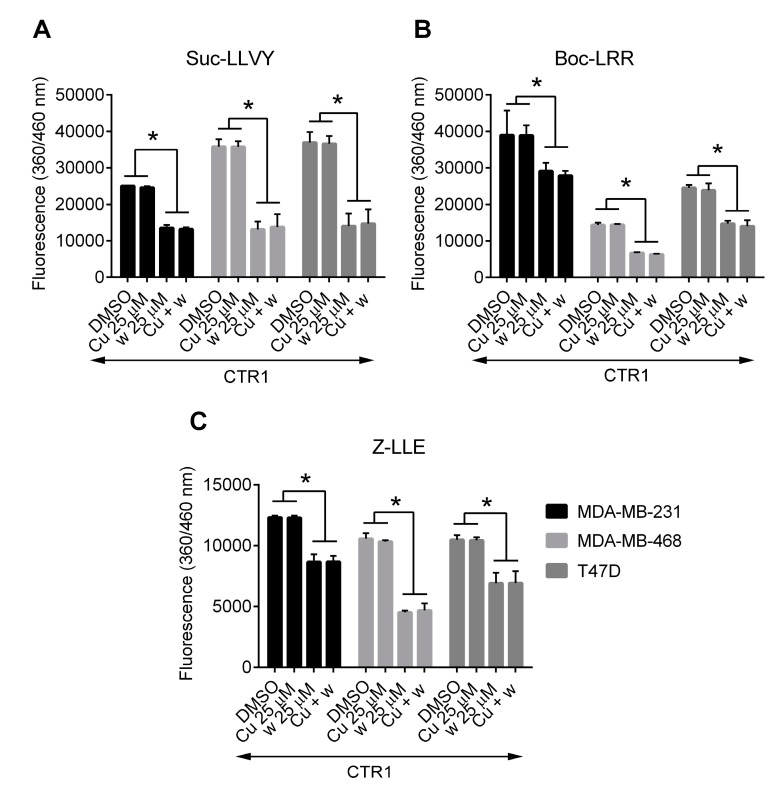
Copper loading does not affect inhibition of proteasome by wedelolactone**.** Cells were transfected with the pCNDA3.1-hCTR1-N-Myc plasmid, loaded with copper (Cu) or left untreated and exposed to various concentrations of wedelolactone (w) or solvent (DMSO) for 10 h. Proteasome activities were evaluated in cell extracts using the activity-specific fluorogenic substrates (Suc-LLVY-AMC for testing chymotrypsin-like (**A**), Z-LLE-AMC for caspase-like (**B**), and Boc-LRR-AMC for trypsin-like activities (**C**)). The data represent the mean values from three independent experiments. Error bars indicate the SD. * indicates a significant (*p* < 0.05) difference.

There are several copper-interacting compounds that have been shown to inhibit proteasome [23,24]. To analyze whether the inhibitory effect of wedelolactone on proteolytic activities of proteasome is also mediated by copper, we compared chymotrypsin-like, trypsin-like and caspase-like proteolytic activities of proteasome in copper-loaded and control breast cancer cells (both overexpressing exogenous *CTR1*). The inhibition of all three proteolytic activities by wedelolactone were found to be similar in copper-loaded and control cells (Figure 5), suggesting that the inhibition of proteasome activities by wedelolactone is a copper-independent process. No significant differences in proteolytic activities were observed in mock-transfected cells that were either pre-incubated with copper or left untreated and subsequently exposed to wedelolactone (Figure S5).

### 2.6. Molecular Docking of Wedelolactone to the Active Sites of Proteasome

To reveal the mechanism of proteasome inhibition by wedelolactone, in silico docking analysis was performed. Since the functional units of proteasome are located at each of the inner β rings, the blind docking was initially performed to assess the specificity of wedelolactone to the active sites. This analysis revealed that wedelolactone occupied the active site of the β5 subunit at least three times more often than the active sites in other units of the protein.

**Figure 6:**
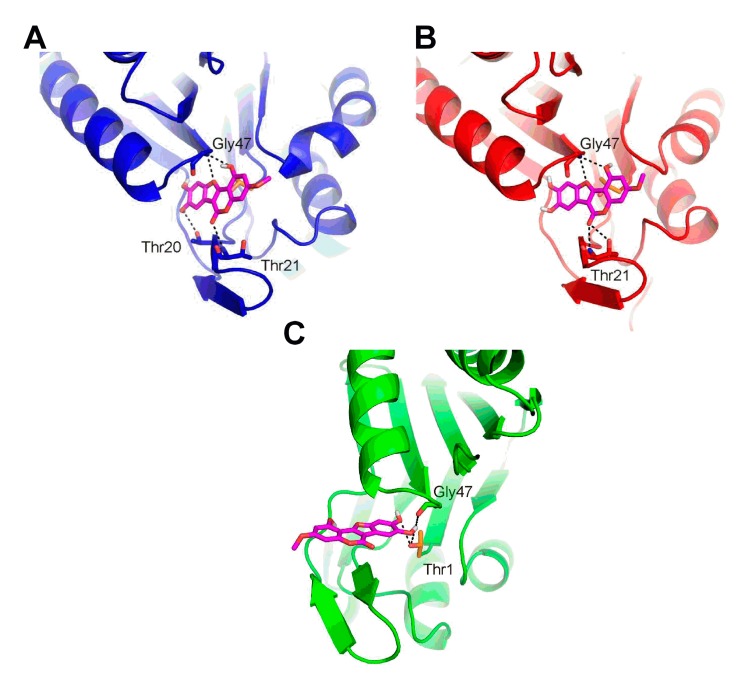
The binding modes of wedelolactone in the β1 (**A**), β2 (**B**), and β5 (**C**) subunits of the yeast 20S proteasome. Wedelolactone is represented as magenta sticks, β1, β2, and β5 subunits are represented as blue, red, and green, respectively. Dashed lines represent the specific H-bonds to the active sites residues. The catalytic residue Thr1 is represented as orange sticks.

The focused docking identified similar binding mode of wedelolactone in the β1 and β2 subunits. Aside from the hydrophobic contacts, wedelolactone formed specific H-bond with the backbone of Thr21 and Gly47 in both subunits. Moreover, wedelolactone formed an additional H-bond with the side-chain of Thr20 in the β1 subunit. Wedelolactone sterically blocked the catalytic residue Thr1 in both β1 and β2 subunits, possibly modulating its proteolytic activity (Figure 6A,B). The focused docking identified a different binding mode of wedelolactone in the β5 subunit. Aside from the specific H-bond with the backbone of Gly47, two hydroxyl groups of wedelolactone were able to form H-bond with the catalytic residue Thr1 (Figure 6C). This specific interaction might be responsible for the preferred binding to the β5 active site over the β1 and β2 active sites, observed in the blind docking. Beside the steric hindrance, the interaction with the catalytic residue Thr1 might be responsible for the elevated inhibition of chymotrypsin-like activity observed experimentally.

## 3. Discussion

Wedelolactone is a natural polyphenolic catechol-type compound with anti-cancer effects that are exerted via multiple mechanisms/targets [12,13,14,15,16,17,18,19,20,21]. In our previous studies, we reported that cytotoxic effect of wedelolactone can be partially attributed to its pro-oxidative and DNA damage activity that is promoted by copper ions. Such activities most likely involve production of ROS and (semi)quinones [22] and were previously described for other polyphenolic compounds [25,26,27]. Very recently, copper-mediated cytotoxicity was confirmed also for coumestrol, another coumestan with structure similar to that of wedelolactone [28]. In accordance with this, cytotoxicity of wedelolactone was enhanced here by copper loading in three breast cancer cell lines. Previously, quinones and ROS, formed by oxidative metabolism of catechol-type polyphenol dopamine, were reported to act as proteasome inhibitors [29]. We found that copper overloading significantly enhanced wedelolactone-induced ROS production and cytotoxicity but it did not further enhance its proteasome inhibitory properties, suggesting the copper-independent mechanism of proteasome inhibition by wedelolactone.

The structural basis of proteasome inhibition by wedelolactone was subsequently revealed by molecular docking. Molecular structure of the core 20S proteasome is extremely conserved and is organized in four stacked rings, each formed by seven subunits in an α7β7β7α7 configuration. Seven distinct β subunits are carrying the enzyme active sites, specifically β1 carries caspase-like activity, β2 is responsible for trypsin-like activity and β5 encodes chymotrypsin-like activity [30]. Protein degradation is facilitated by nucleophilic N-terminal threonine (Thr1) residues of catalytic β subunits, in which the side chain hydroxyl group reacts with peptide bonds of substrates as well as functional groups of inhibitors [31]. Inhibitors of the 20S proteasome can be divided into two main groups based on whether or not they form a covalent bond with the active site Thr1 according to classification proposed by Kisselev et al. [10]. Molecular docking revealed that wedelolactone occupies the active sites of β1, β2, and β5 proteasomal subunits. While similar binding mode was predicted for β1 and β2 subunit, a specific interaction between both hydroxyl groups of wedelolactone with catalytic residue Thr1 was observed only in β5 subunit. These differences in binding modes are probably responsible also for predicted favored interaction with β5 subunit and might explain a stronger inhibition of proteasomal chymotrypsin-like activity compared to trypsin- and caspase-like activities.

The observed IC_50_ values for chymotrypsin-like inhibitory activity of wedelolactone were below 10 μM in vitro and within 10–25 μM range in cells. It is noteworthy that wedelolactone induced growth arrest and apoptosis in all three breast cancer cell line tested at concentrations corresponding to the above mentioned IC_50_ values [12,22]. This suggests that inhibition of proteasome may contribute significantly to cytotoxicity of this compound.

Inhibition of proteasome results in increased levels of polyubiquitinated proteins because most of the proteasome-mediated protein degradation pathways require ubiquitination [32]. Moreover proteins with high turnover, including p21, p27, p53, and Bax accumulates in cells in response to proteasomal inhibition [33,34,35]. Such accumulation was clearly documented here in wedelolactone-treated cells. It is important to note that some previous studies showing connection between the treatment with wedelolactone and altered expression of numerous proteins should be interpreted with caution as wedelolactone can affect not only protein expression but also protein degradation pathway.

This study concluded that natural coumestan wedelolactone acts as a copper-independent proteasome inhibitor with potency similar to other flavonoids. As cancer cells were reported to be more sensitive to proteasome inhibition, this novel function of wedelolactone might explain its preferred toxicity towards cancer cells observed previously [20]. Understanding a structural basis for inhibitory mode of wedelolactone might help to open up new avenues for design of novel compounds efficiently inhibiting cancer cells.

## 4. Material and Methods

### 4.1. Chemicals and Plasmids

Chemicals were obtained from commercial providers: wedelolactone, dimethyl sulfoxide (DMSO), propidium iodide (PI), and copper sulfate (Sigma-Aldrich, St. Louis, MO, USA), dihydroethidium (DHE; Cayman Pharma, Ann Arbor, MI, USA), Proteasome Activity Fluorometric Assay Kit II (UPBio, Aurora, CO, USA). The pCNDA3.1-hCTR1-N-Myc plasmid was kindly provided by Dennis J. Thiele [36].

### 4.2. Cell Culture

The human breast cancer cell lines MDA-MB-231, MDA-MB-468, and T47D were cultured in HEPES-modified RPMI 1640 medium (Sigma-Aldrich) supplemented with 10% fetal calf serum (FCS, Sigma-Aldrich), 2 mM l-glutamine, 100 U/mL penicillin, and 100 μg/mL streptomycin (Lonza, Verviers, Belgium) in a humidified atmosphere of 5% CO_2_ at 37 °C. In all experiments, wedelolactone was applied at concentrations that have been shown previously to effectively induce cell death in breast cancer cell lines [12,22].

### 4.3. Proteasome Activity Assay

#### 4.3.1. Purification of 26S Proteasome from MDA-MB-231 Cells

Human 26S proteasome was purified from 8 × 10^6^ of MDA-MB-231 cells using The Rapid 26S Proteasome Purification Kit (J4310, UBPBio) according to manufacturer’s instructions.

#### 4.3.2. Proteasome Activity In Vitro

Chymotrypsin-like, trypsin-like and caspase-like proteasome activities were determined using Proteasome Activity Fluorometric Assay Kit II (J4120, UBPBio) according to manufacturer’s instructions. Briefly, wedelolactone was added at various concentrations to 150 μL reaction mixture containing either purified 10 nM bovine 20S proteasome (A1400, UBPBio) or 5 μg of MDA-MB-231-purified 26S proteasome complex, and 50 μM fluorogenic substrate (Suc-LLVY-AMC to test chymotrypsin-like activity, Z-LLE-AMC to test caspase-like activity and Boc-LRR-AMC to test trypsin-like activity) in 1× Proteasome Assay Buffer (40 mM Tris, pH 7.1, 2 mM β-mercaptoethanol; UBPBio). MG132 at concentration of 10 μM and aliquots of DMSO were used as positive and negative controls, respectively. Fluorescence was measured by TECAN infinite 200 plate reader (TECAN, Mannedorf, Switzerland) for 1 h at 37 °C.

#### 4.3.3. Proteasome Activity in Cancer Cell Lines

MDA-MB-231, MDA-MB-468 and T47D (6 × 10^5^) cells were seeded in 5 mL of culture media, exposed to various concentrations of wedelolactone, DMSO or 10 μM MG132 for 10 h. Cells were harvested, resuspended in cell lysis buffer (40 mM Tris, pH 7.2, 50 mM NaCl, 2 mM β-mercaptoethanol, 2 mM ATP, 5 mM MgCl, 10% Glycerol) and briefly sonicated using an Ultrasonic Processor UP100H (Hielscher, Ringwood, NJ, USA). Cell lysates were cleared by centrifugation and protein concentration in supernatant was determined using DC protein assay (Biorad, Hercules, CA, USA). Protein extract (50 μg) was mixed with 50 μM of fluorogenic substrates (UBPBio) in 1× Proteasome Assay Buffer in a total volume of 100 μL. Fluorescence was measured by TECAN infinite 200 plate reader (TECAN) for 1 h at 37 °C.

#### 4.3.4. Proteasome Activity after Copper-Overloading

MDA-MB-231, MDA-MB-468, and T47D cells (6 × 10^5^) were seeded in 5 mL of culture media. Next day, transient transfection was performed, using 4 μL of Lipofectamine LTX reagent (Invitrogen, Carlsbad, CA, USA) with a mixture containing 2 μg of pCDNA3.1-hCTR1-N-Myc or control pCDNA3.1 plasmid and 2 μL of PLUS reagent (Invitrogen). Six hours later, the medium was replaced, cells were treated with 25 μM copper sulfate or left untreated for 24 h, and then were exposed to wedelolactone or DMSO for 10 h in fresh media. Cells were then harvested and proteasome activity was analyzed as described in 4.3.3.

### 4.4. Immunoblotting

5 × 10^5^ cells were seeded in 6-well plates. The next day, the cells were exposed to various concentrations of wedelolactone, DMSO, or 10 μM MG132 for 10 h. Cells were harvested and lysed as described previously [37]. Cell lysates were subjected to SDS–PAGE and immunoblotted. Sample loading was normalized according to protein concentration determined by DC protein assay (Biorad). Blots were probed with anti-ubiquitin (3933S; Cell Signaling Technology, Inc., Beverly, MA, USA), anti-p21, anti-p27 (sc-817 and sc-528; Santa Cruz Biotechnology Inc., Santa Cruz, CA, USA), anti-p53, anti-Bax or anti-Myc-Tag (9282, 5023 and 2276S; Cell Signaling Technology), anti-α-tubulin antibodies (T9026; Sigma-Aldrich), and secondary antibodies conjugated with peroxidase (Sigma-Aldrich). Blots were developed with a standard ECL procedure with Immobilon Western Chemiluminiscent HRP Substrate (Millipore, Billerica, MA, USA).

### 4.5. RNA Isolation, cDNA Synthesis and qPCR

1 × 10^6^ cells were seeded in 5 mL dishes. Next day, the cells were exposed to various concentrations of wedelolactone or DMSO for 10 h. Cells were harvested and total RNA was isolated using GenElute Mammalian Total RNA Miniprep kit (Sigma-Aldrich). For cDNA synthesis, 1 μg of total RNA was reverse-transcribed using the QuantiTect Reverse Transcription kit (Qiagen, Hilden, Germany) according to the manufacturer’s instructions in a final reaction volume of 20 μL. Expression of *CDKN1A*, *CDKN1B*, *TP53* and *BAX* genes was determined using the target-specific primers (Table S1) and KAPA SYBR FAST qPCR MASTER MIX (KK460, Kapa Biosystems, Cambridge, MA, USA) on LightCycler 480 II (Roche, Basel, Switzerland). Expression of the reference *GAPDH* gene (probe 4326317E, ThermoFisher Scientific, Waltham, MA, USA) was used for data normalization.

### 4.6. Cell Mortality

3 × 10^5^ cells were seeded in 6-well plates. After 24 h, transient transfection was performed, using 3 μL of Lipofectamine LTX reagent (Invitrogen) with a mixture containing 1.5 μg of pCNDA3.1-hCTR1-N-Myc or control pCDNA3.1 plasmid and 1.5 μL of PLUS reagent (Invitrogen). Six hours later, the medium was replaced, cells were either exposed to 25 μM copper sulfate for 24 h or left untreated and subsequently subjected to wedelolactone or DMSO for 48 h in fresh media. Cytotoxicity of wedelolactone was analyzed 48 h later by PI staining (1 μg/mL) using flow-cytometry as described previously [38].

### 4.7. Reactive Oxygen Species Production Analysis

3 × 10^5^ cells were seeded in 6-well plates. After 24 h, transient transfection was performed as described in chapter 4.6. Medium was replaced after 6 h, cells were left untreated or pretreated with 25 μM copper sulfate for 24 h, and subsequently exposed to wedelolactone or DMSO for 10 h in fresh media. The cells were washed with PBS and stained with 10 μM DHE for 20 min at 37 °C in the dark. Reactive oxygen species (ROS) were measured using flow-cytometry (BD FACSVerse, BD Biosciences, Franklin Lakes, NJ, USA) at an excitation wavelength of 485 nm and an emission wavelength of 538 nm. Data were analyzed using BD FACSuite software (BD Biosciences).

### 4.8. Analysis of Copper Concentrations in Cells

For detection of intracellular copper concentrations, 6 × 10^5^ cells were seeded in 5 mL of growth medium. The next day, the cells were transiently transfected with CTR1 or control pCDNA3.1 vector. Transfection was performed using 4 μL of the Lipofectamine LTX reagent (Invitrogen) with a mixture containing 2 μg of plasmid and 2 μL of PLUS reagent (Invitrogen). Six hours later, the medium was replaced with fresh one and cells were pretreated with 25 μM copper sulfate or left untreated for 24 h. Then, the cells were exposed to 25 μM wedelolactone or DMSO for 10 h in fresh media. Cells were harvested and cell lysates prepared as described previously [39]. Briefly, pelleted cells were washed twice with 1 × PBS and 1.0 × 10^6^ cell were lysed in a mixture of 3 M HCl/10% trichloroacetic acid at room temperature for 3 h followed by incubation at 70 °C for 5 h. The lysate was centrifuged (600 g/5 min) to remove cell debris and the total amount of copper in supernatant was determined by substrate-assisted laser desorption inductively-coupled plasma mass spectrometry (SALD ICP MS) [40].

Each sample was mixed with 400 μg/L aqueous solution containing nickel as an internal standard (ASTASOL-^®^Ni, CRM, ANALYTIKA^®^, Prague, Czech Republic) in the 1:1 ratio and spotted by a micropipette onto a polyethylene terephthalate plate (PET) as a 200 nL droplet in seven replicates. The sample plate was inserted into an ablation system (model UP 213, New Wave, Fremont, CA, USA) and spots were scanned by an Nd:YAG 213 nm laser beam in a zig-zag shaped raster with the raster spacing 190 μm; the laser beam waist was adjusted to the size ~250 μm. Size of the raster was selected according to the spot diameter to desorb the entire sample (typical spot diameter ~1.4 mm), and the analysis time of each sample was approximately 2 min. The ablation cell was flushed with a carrier gas (helium, flow rate 1.0 L/min), which transported the aerosol to an ICP mass spectrometer (model 7500CE, Agilent Technologies, Santa Clara, CA, USA). A sample gas flow of argon was admixed to the helium carrier gas flow subsequent to the laser ablation cell (0.6 L/min). Optimization of LA ICP MS conditions (gas flow rates, sampling depth, electrostatic lens voltages of the MS) was performed with the glass reference material NIST SRM 612 regarding the maximum signal-to-noise ratio and minimum oxide formation (ThO^+^/Th^+^ counts ratio 0.2%, U^+^/Th^+^ counts ratio 1.1%). Other ICP MS parameters were adjusted in compliance with the manufacturer’s recommendations. The laser fluence was ~0.75 J/cm^2^, the repetition rate 10 Hz, and the scan rate 160 μm/s. The ions were measured with an integration time 0.1 s. Both the flush time and the laser warm-up time were set to 10 s. The ion signal of two copper isotopes, ^63^Cu and ^65^Cu, was monitored to reveal possible polyatomic interferences. The signal ratio of the most abundant isotopes, ^63^Cu and ^60^Ni as the internal standard was used for data evaluation.

### 4.9. Molecular Docking

The three-dimensional structure of wedelolactone was downloaded from ZINC database [41], (ZINC ID: ZINC6483512). The output file in Sybyl mol2 format was converted into AutoDock Vina [42] compliant pdbqt format by MGLTools [43]. The crystal structure of yeast 20S proteasome (PDB ID: 5CZ4) was used as a target in molecular docking. All ligands and water molecules were removed from the target molecule. The hydrogen atoms were added to the target by PyMol [44]. The Gasteiger charges and AutoDock atom types were assigned to targets by MGLTools. The active site of β1 (caspase-like activity), β2 (trypsin-like activity) and β5 (chymotrypsin-like activity) subunits and both inner β rings were selected as target regions for molecular docking performed by AutoDock Vina. The region selected for focused docking was represented by a box of 22.5 Å × 22.5 Å × 22.5 Å centered at the catalytic residue Thr1. The entire protein surface was selected for a blind docking to assess the specificity of wedelolactone towards the enzyme active sites. The region selected for the blind docking was represented by a box with 87.5 Å × 87.5 Å × 87.5 Å dimension centered at the middle of the two inner β rings harboring the active sites. Ten and twenty conformations were produced by AutoDock Vina in the focused and blind docking, respectively. The docked conformations were re-scored by NNScore 2.0 [45], which predicts binding affinity of the conformation as an average over 20 distinct neural-networks.

### 4.10. Statistics

Values were expressed as means ± standard deviations (SD). To determine statistical significance, the values were compared using a two-tailed *t*-test for unpaired samples. Differences were considered to be statistically significant with the *p*-value < 0.05. IC_50_ values were determined by nonlinear regression using GraphPad PRISM 6 software (GraphPad-San Diego, CA, USA). All results were reproduced at least in three independent experiments.

## Supplementary Materials

Supplementary materials can be found at www.mdpi.com/1422-0067/18/4/729/s1.

## Abbreviations

DHE	Dihydroethidium
DMSO	Dimethyl sulfoxide
ECL	Enhanced chemiluminescence
FCS	Fetal calf serum
CTR1	Human copper transporter 1
PDB	Protein data bank
PI	Propidium iodide
ROS	Reactive oxygen species
SALD ICP MS	Substrate-assisted laser desorption inductively-coupled plasma mass spectrometry
SDS-PAGE	Sodium dodecyl sulfate polyacrylamide gel electrophoresis
qPCR	Quantitative polymerase chain reaction
UPS	Ubiquitin-proteasome system
