# Supplementary material for: Wedelolactone Acts as Proteasome Inhibitor in Breast Cancer Cells
Source: Int J Mol Sci. 2017 Mar 29;18(4):729. doi: 10.3390/ijms18040729 (PMC5412315; doi:10.3390/ijms18040729)
Supplement: Supplementary file 1 [file ijms-18-00729-s001.pdf]

Wedelolactone Acts as Proteasome Inhibitor in Breast Cancer Cells

Tereza Nehybová, Jan Šmarda, Lukáš Daniel, Marek Stiborek, Viktor Kanický, Ivan Spasojevič, Jan Preisler, Jiří Damborský and Petr Beneš

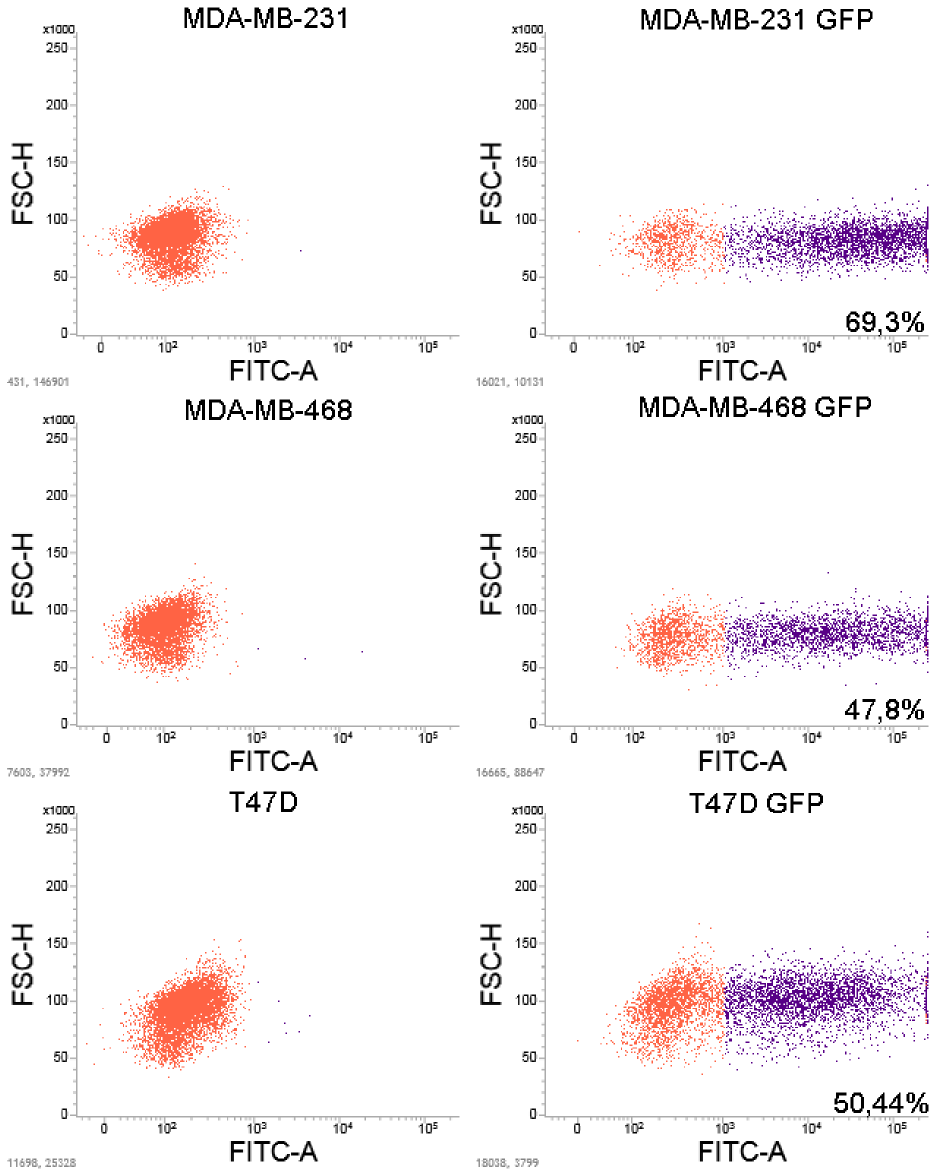

Figure S1. Efficiency of transient transfection in breast cancer cell lines. MDA-MB-231, MDA-MB-468, and T47D cells (6×10^5) were seeded in 5 ml of culture media. The next day, transient transfection was performed, with a mixture containing 2 µg of pEGFP-C1 plasmid (Clontech, Mountain View, CA, USA) and 2 µl of PLUS reagent (Invitrogen, Carlsbad, CA, USA) using 4 µl of LipofectamineLTX reagent (Invitrogen). Six hours later, the medium was replaced and cells were cultivated for additional 48 hours. Green fluorescence positivity was evaluated by flow-cytometry (BD FACSVerse, BD Biosciences, Franklin Lakes, NJ, USA) at an excitation wavelength of 485 nm and an emission wavelength of 538 nm. Data were analyzed using BD FACSuite software. The representative dot plots are presented.

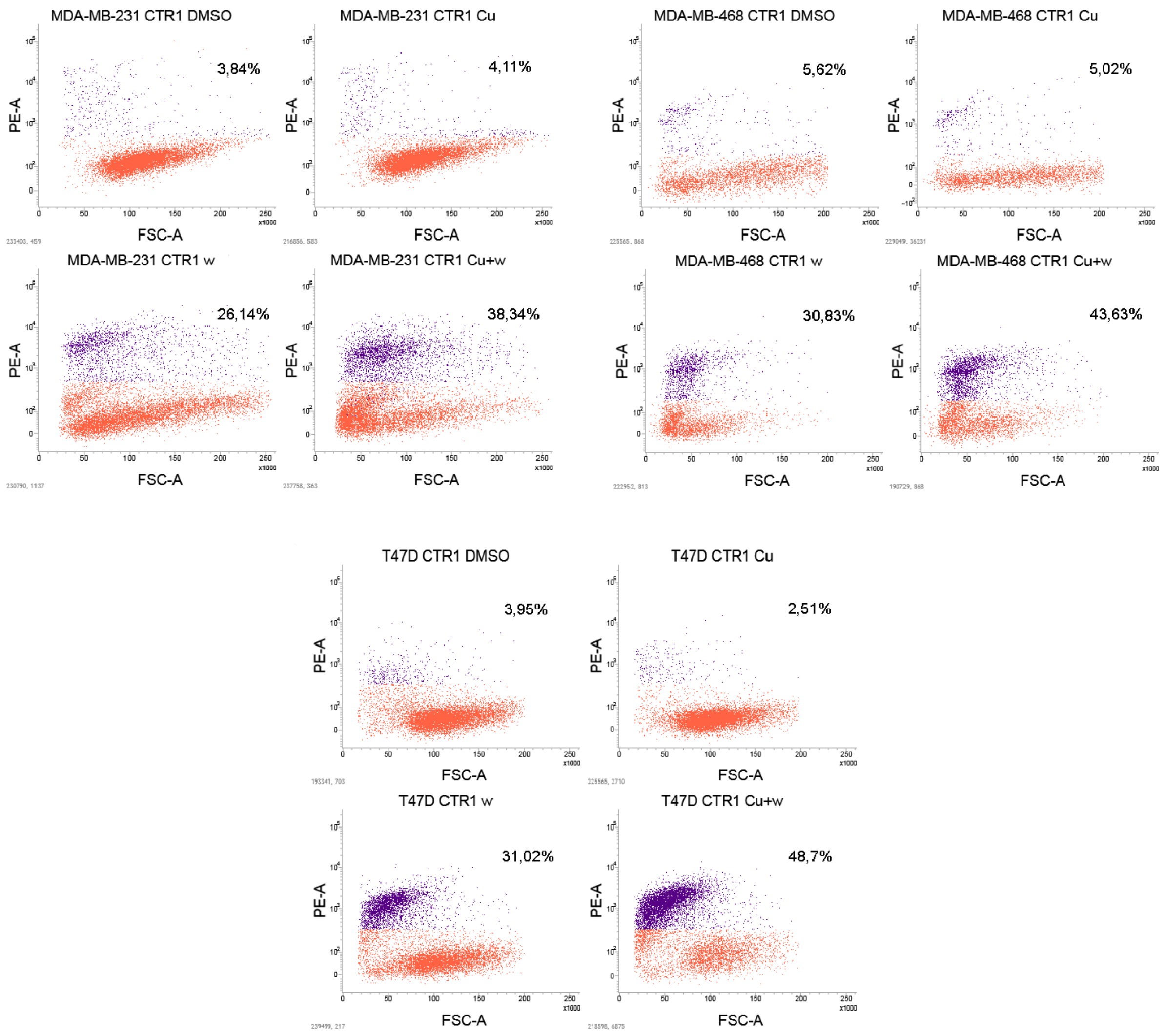

Figure S2. Copper loading enhances cytotoxicity of wedelolactone to breast cancer cells. MDA-MB-231, MDA-MB-468, and T47D cells were transiently transfected with pCNDA3.1-hCTR1-N-Myc (CTR1), pretreated with copper sulfate (Cu) for 24 h and subsequently treated with wedelolactone (w) or solvent (DMSO) in fresh media. Cell mortality was evaluated after PI staining using flow-cytometry. The representative dot plots are presented.

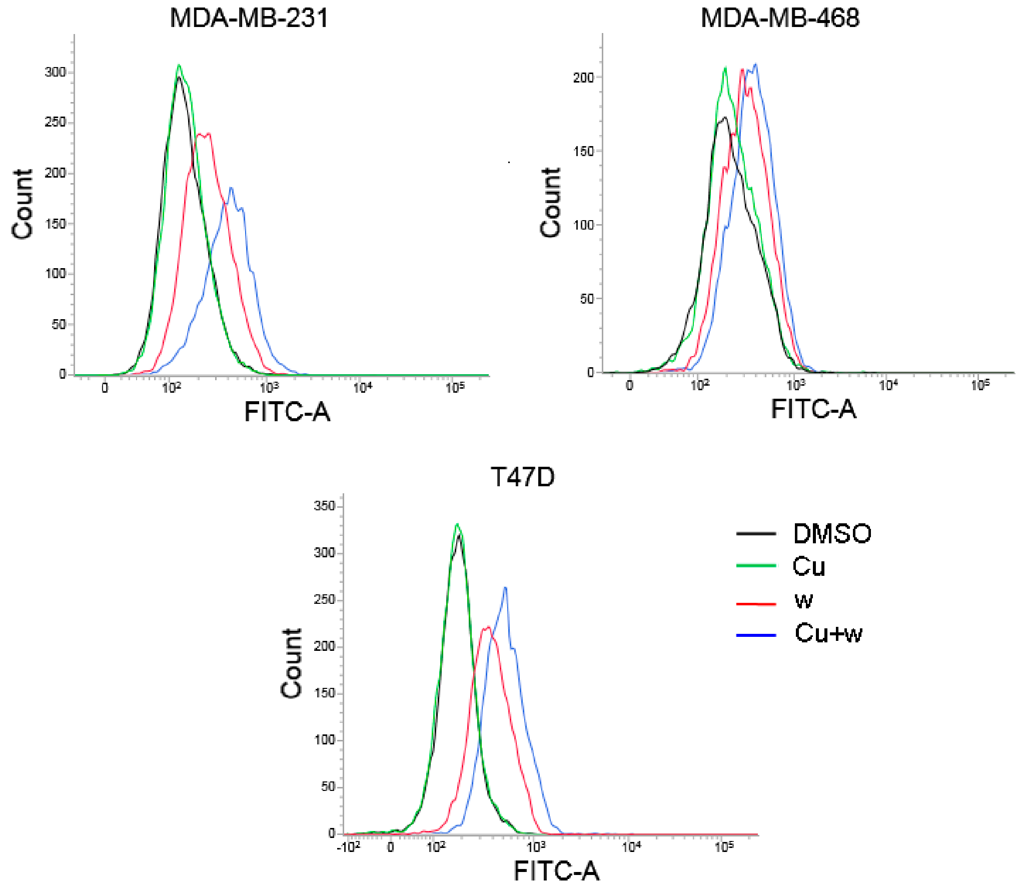

Figure S3. Copper loading enhances ROS production in the wedelolactone-treated breast cancer cells. MDA-MB-231, MDA-MB-468, and T47D cells were transiently transfected with pCNDA3.1-hCTR1-N-Myc (CTR1), pretreated with copper sulfate (Cu) for 24 h and subsequently treated with wedelolactone (w) or solvent (DMSO) in fresh media. Reactive oxygen species (ROS) production was evaluated after dihydroethidium (DHE) staining using flow-cytometry. The representative histograms are presented.

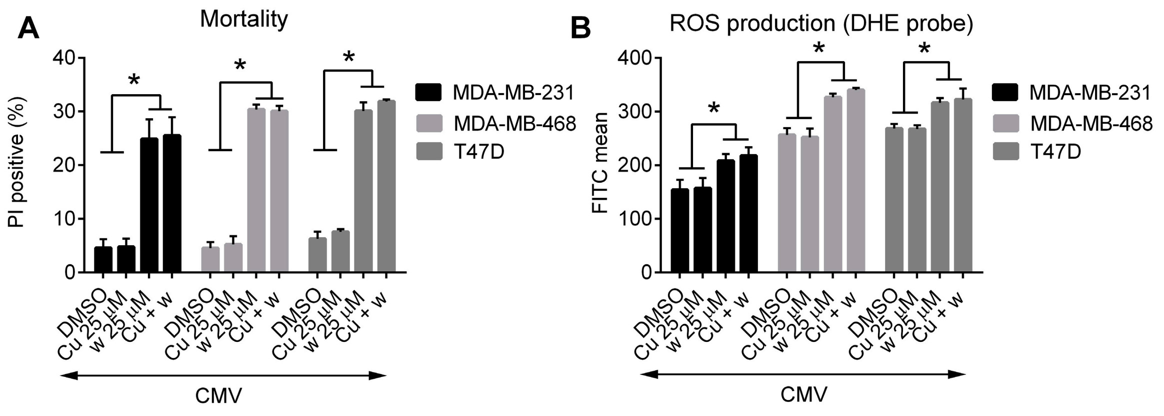

Figure S4. Pretreatment with copper does affect neither mortality nor ROS production of mock-transfected cells exposed to wedelolactone or dimethyl sulfoxide (DMSO). Cells were transiently transfected with control pCDNA3.1 (cmv) plasmid, pretreated with copper sulfate (Cu) or left untreated for 24 h and subsequently exposed to wedelolactone (w) or solvent (DMSO) in fresh media. Cells were harvested, (A) cell mortality and (B) ROS production was evaluated after propidium iodide/DHE staining using flow-cytometry. The data represent the mean values from three independent experiments. Error bars indicate the SD. * indicates a significant ($p < 0.05$) difference.

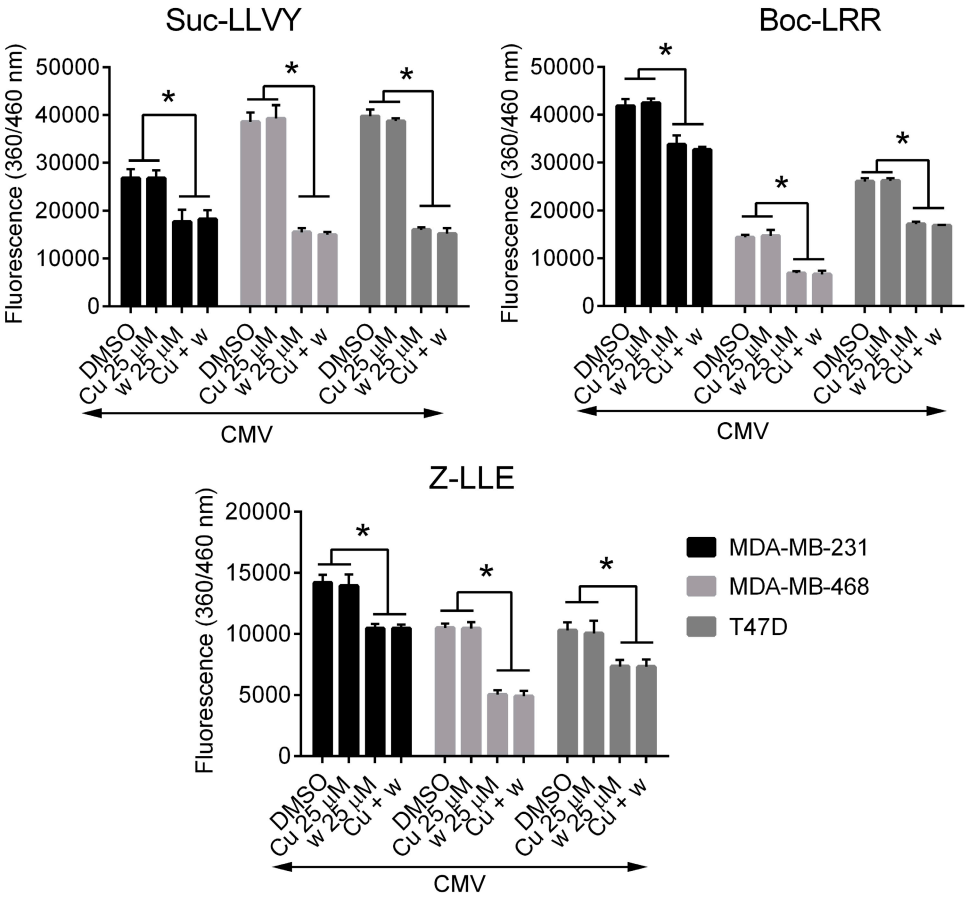

Figure S5. Pretreatment with copper does not affect proteasome activities of mock-transfected cells exposed to wedelolactone or DMSO. Cells were transfected with control pCDNA3.1 (cmv) plasmid, pretreated with copper sulfate (Cu) for 24 h or left untreated and exposed to various concentrations of wedelolactone (w) or solvent (DMSO) for 10 h. Proteasome activities in cell extracts were subsequently analysed using fluorogenic substrates (Suc-LLVY-AMC, Z-LLE-AMC or Boc-LRR-AMC). The data represent the mean values from three independent experiments. Error bars indicate the SD. * indicates a significant ($p < 0.05$) difference.

Table S1. Primer sequences for qPCR.

CDKN1A	5′-GCA TGA CAG ATT TCT ACC ACT CCA-3′
	5′-GCA GAA GAT GTA GAG CGG GC-3′
CDKN1B	5′-GCT AAC TCT GAG GAC ACG CA-3′
	5′-TAG AAG AAT CGT CGG TTG CAG G-3′
TP53	5′-TAA CAT GGA GCT GCA GAG GAT G-3′
	5′-GGG ACA TCA GTC GCT TCA GTG-3′
BAX	5′-ACC TAT GGA AAC TAC TTC CTG AAA-3′
	5′-CTG GCA TTC TGG GAG CTT CA-3′
